# Pulmonary Vein Thrombus After Mitral Valve Transcatheter Edge-to-Edge Repair

**DOI:** 10.14797/mdcvj.1428

**Published:** 2024-08-12

**Authors:** Aakash Rana, Jack Xu, Jedidiah McMunn, Rupesh Manam

**Affiliations:** 1 Central Arkansas Veterans Affairs Health System, Little Rock, Arkansas, US; 2 Novant Health Forsyth Medical Center, Winston-Salem, North Carolina, US

**Keywords:** pulmonary vein thrombus, mitraclip, mitral valve, transcatheter edge-to-edge repair

## Abstract

A 76-year-old female with a complicated medical history presented for Watchman (Boston Scientific) placement 2 months after mitral valve transcatheter edge-to-edge repair (TEER). Preoperative workup before Watchman placement confirmed the presence of a thrombus in the left superior pulmonary vein. Post-procedure mitral valve TEER transesophageal echocardiogram showed no thrombus in the left atrium appendage or pulmonary veins. We believe the thrombus in the left superior pulmonary vein occurred secondarily due to epithelium damage during the mitral valve TEER.

## Introduction

Pulmonary vein thrombosis (PVT) is a rare entity that is underdiagnosed in clinical practice. Common causes of PVT include surgeries involving the pulmonary vein such as lung transplant, lobectomy, and radiofrequency catheter ablation for atrial fibrillation as well as infiltration or compression of the pulmonary vein by primary or secondary lung tumors.^1^,^2^ Patients with PVT are usually asymptomatic or have symptoms of dyspnea, cough, or hemoptysis.^3^ Diagnosis of PVT requires a combination of imaging modalities such as transthoracic echocardiography, transesophageal echocardiography (TEE), computed tomography (CT), and magnetic resonance imaging to distinguish between tumor and thrombus.^1^,^4^,^5^,^6^ Determining the treatment for PVT depends on the underlying obstructive pathology and may involve options such as antibiotic therapy, anticoagulation, thrombectomy, and/or pulmonary resection.^7^ We present a rare case of PVT in the left upper pulmonary vein after mitral valve transcatheter edge-to-edge repair (TEER).

## Case Presentation

A 76-year-old female presented for Watchman (Boston Scientific) placement 2 months after mitral valve transcatheter edge-to-edge repair (TEER). Her past medical history included paroxysmal atrial fibrillation status post pulmonary vein isolation ablation on apixaban, severe mitral regurgitation status post mitral valve TEER, moderate tricuspid regurgitation, mild aortic stenosis, coronary artery disease, heart failure with preserved ejection fraction, hypertension, and renal artery stenosis status post stenting of right kidney and Stage 3a chronic kidney disease. The patient had been on rivaroxaban for years with no missed doses before the procedure. Blood pressure (BP) was mildly elevated (165/75 mm Hg) with other stable vital signs. Labs and physical examination were unremarkable. Transesophageal echocardiography with color Doppler showed an echogenic mass within the left superior pulmonary vein, suggestive of a thrombus (Videos 1 and 2).

**Video 1:** Transesophageal echocardiography showing a thrombus in the left upper pulmonary vein in the presence of MitraClip; see also at https://youtu.be/ukYRitIx3_8.

**Video 2:** Transesophageal echocardiography with color Doppler showing a thrombus in the left upper pulmonary vein in the presence of MitraClip; see also at https://youtu.be/NsPPSGp1BiI.

**Figure 1:**
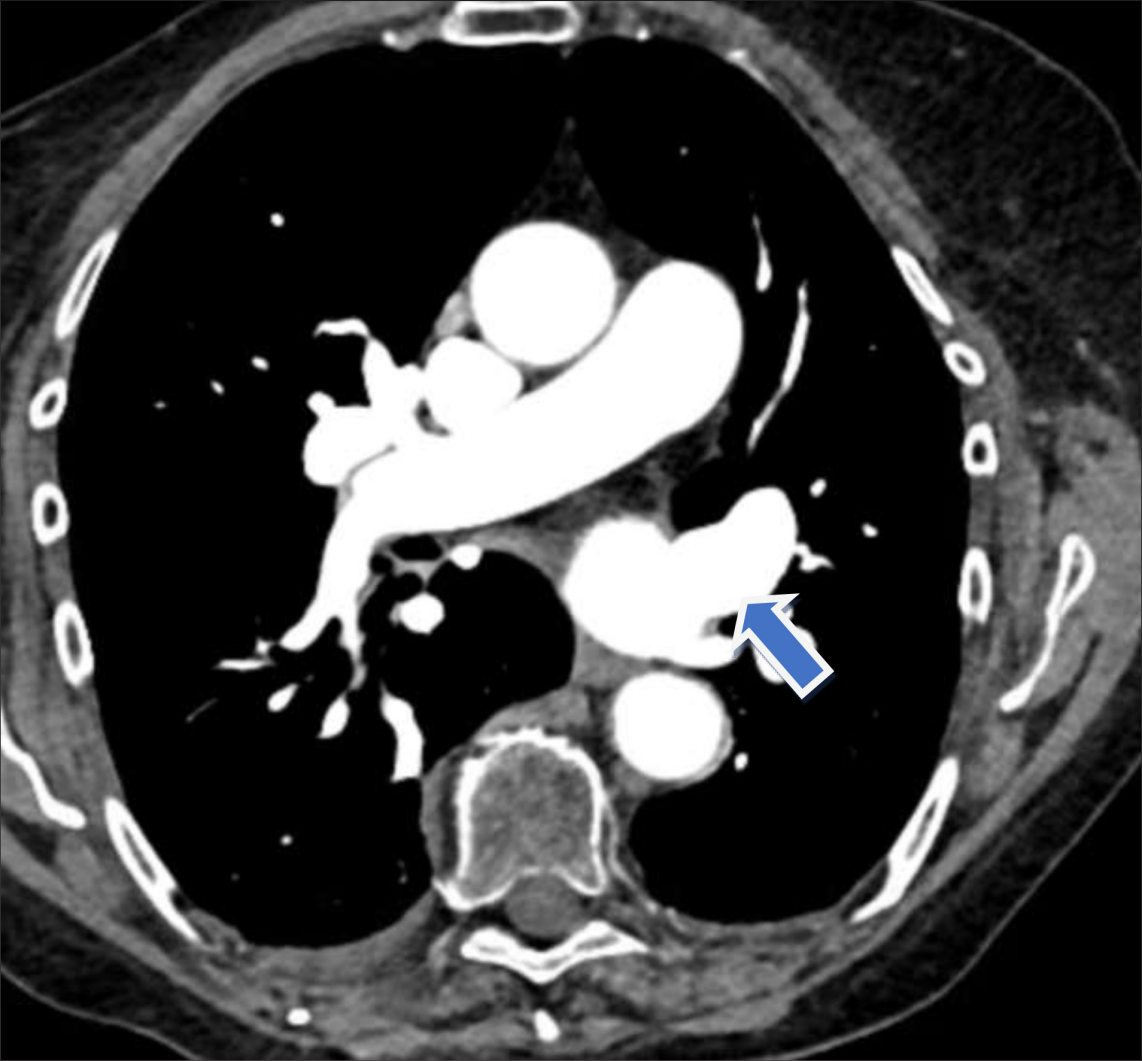
Computed tomography of the chest showing no thrombus in the left upper pulmonary vein.

Watchman placement was aborted, and the patient was switched from rivaroxaban to apixaban as there was concern that the patient was a nonresponder to rivaroxaban. The TEE prior to mitral valve TEER showed severe mitral regurgitation (2-3+), severe tricuspid regurgitation, and no thrombus in the left atrium appendage or pulmonary veins. Rivaroxaban was paused for 3 days before mitral valve TEER. Post-procedure mitral valve TEER TEE showed reduction of mitral regurgitation to 1+ from 2-3+ with proper deployment of MitraClip (Abbott), mean mitral valve gradient 2 mm Hg, and no thrombus in the left atrium appendage or pulmonary veins. A chest CT obtained 6 weeks after initiation of apixaban showed resolution of the thrombus in the left superior pulmonary vein (Figure 1). Timeline of events relating to the case is shown in Table 1.

**Table 1:** Case timeline. TEER: transcatheter edge-to-edge repair; X: day of mitral valve TEER; TEE: transesophageal echocardiogram; MR: mitral regurgitation; TR: tricuspid regurgitation; CT: computed tomography


CASE TIMELINE	

X – 48 months	Underwent pulmonary vein isolation ablation for atrial fibrillation

X – 2 months	TEE before mitral valve TEER showed severe MR (2–3+), severe TR, and no thrombus in the left atrium appendage or pulmonary veins

X – 3 days	Rivaroxaban was paused for 3 days before mitral valve TEER

X	Underwent mitral valve TEER for severe MR. TTE after mitral valve TEER showed a reduction of MR (2–3+ to 1+), mean mitral valve gradient 2 mm Hg, no thrombus in the left atrium appendage or pulmonary veins. Rivaroxaban resumed the next day

X + 2 months	Day of Watchman placement: TEE showed a thrombus in the left superior pulmonary vein. Watchman placement was aborted, and the patient was switched from rivaroxaban to apixaban

X + 3.5 months	CT of the chest showed resolution of the thrombus in the left superior pulmonary vein while on apixaban for 6 weeks


## Discussion

Pulmonary vein thrombus is a rare entity and often underdiagnosed. It usually occurs due to the tumor extending directly into the vein, the tumor pressing on the vein, damage to the epithelium, or increased blood clotting.^8^,^9^ In our patient, TEE prior to and after mitral valve TEER showed no thrombus in the left superior pulmonary vein. When the patient presented for Watchman placement 2 months after mitral valve TEER, a thrombus was present in the left superior pulmonary vein on routine pre-op TEE. We believe the thrombus in the left superior pulmonary vein occurred secondarily due to epithelium damage during the mitral valve TEER.

During mitral valve TEER, a transeptal puncture is performed to access the left atrium. An Amplatz (Boston Scientific) extra stiff wire is passed via the interatrial septum and placed in the left upper pulmonary vein while the steerable guide catheter with dilator is advanced over the guidewire into the left atrium.^10^ After securely positioning the catheter in the left atrium, the Amplatz extra stiff wire is withdrawn, followed by the dilator. Under fluoroscopic guidance, the clip delivery system is maneuvered through the steerable guide catheter towards the left upper pulmonary vein followed by grasping of the mitral leaflets and clip release.^10^ Mitral valve TEER can lead to a degree of iatrogenic mitral stenosis, which can lead to slower flow in the left atrium and pulmonary veins. However, mean mitral valve gradient after mitral valve TEER was 2 mm Hg in this case.

In our patient, the PVT was found incidentally on routine preprocedure TEE for Watchman placement. As cited in the literature, most PVT cases are asymptomatic and are only detected incidentally through imaging.^3^ Various treatments have been documented for PVT, such as antibiotic therapy, anticoagulation, surgical removal of the thrombus, and pulmonary resection. Our patient was hemodynamically stable without hemoptysis, pulmonary gangrene, or necrosis. The patient was successfully treated with apixaban for 6 weeks with repeat imaging showing resolution of the PVT. It is essential to emphasize that prior literature has not conducted comparisons between different anticoagulant options and their durations. Therefore, additional data is needed to assess the risks and benefits associated with these choices.^11^

## Conclusion

In conclusion, PVT is increasingly being detected due to the rising frequency of various imaging modalities. Nevertheless, recognizing PVT as a potential complication of mitral valve TEER can aid in minimizing delays in diagnosis and treatment. Addressing the risk of systemic embolization with appropriate anticoagulation is crucial.
